# Immediate Effects of the Forehead Exercise for Suprahyoid Muscles: A Case Series

**DOI:** 10.7759/cureus.87347

**Published:** 2025-07-05

**Authors:** Kazuya Hara, Takashi Shigematsu, Keishi Okamoto, Kenjiro Kunieda, Tomohisa Ohno, Ichiro Fujishima

**Affiliations:** 1 Department of Rehabilitation Medicine, Hamamatsu City Rehabilitation Hospital, Shizuoka, JPN; 2 Department of Neurology, Gifu University Graduate School of Medicine, Gifu, JPN; 3 Department of Dentistry, Ryohoku Hospital, Tokyo, JPN

**Keywords:** dysphagia, isometric exercise, suprahyoid muscles, swallowing rehabilitation, videofluoroscopic swallowing study

## Abstract

Introduction

Dysphagia is common in older adults and individuals with neurological disorders and is often associated with aspiration pneumonia and reduced quality of life. The Forehead Exercise for Suprahyoid Muscles (FESM, also known as Enge-Odeko-Taiso) is a simple, non-invasive technique aimed at strengthening the suprahyoid muscles. However, its immediate effect on swallowing function is not well understood. We aimed to determine whether a single session of FESM, performed during a videofluoroscopic swallowing study (VFSS), could immediately reduce the risk of penetration and aspiration, as measured by the Penetration-Aspiration Scale (PAS).

Methods

This small retrospective case series included 12 patients (mean age 76.6 ± 11.3 years) who underwent VFSS at Hamamatsu City Rehabilitation Hospital between December 1, 2024, and April 1, 2025. Patients were included if they had a baseline PAS score ≥2 and performed the FESM during the same VFSS session. Patients performed at least five repetitions of the FESM, which involved isometric chin-tuck contractions, immediately before the post-intervention swallow. Body position, bolus type, and imaging parameters were kept consistent before and after the exercise. Pre- and post-FESM PAS scores were compared using the Wilcoxon signed-rank test (significance set at p < 0.05).

Results

The median PAS score significantly decreased from 3.5 (interquartile range (IQR), 2.75-8.00) to 1.0 (IQR, 1.00-2.25) following FESM (p = 0.025). Among the 12 patients, 10 showed improvement, one showed no change, and one worsened.

Conclusion

A single FESM session performed immediately prior to swallowing significantly reduced penetration and aspiration events. These findings suggest that FESM may be a practical, low-effort preparatory exercise to reduce the risk of airway invasion during swallowing. Further controlled trials are needed to confirm these effects and to investigate potential dose-response relationships and long-term benefits.

## Introduction

Dysphagia is a prevalent clinical condition, particularly among older adults and individuals with neurological disorders. It significantly impairs quality of life and is associated with increased risks of aspiration pneumonia [1,2] and malnutrition [3]. A key contributor to dysphagia is weakness in the suprahyoid and tongue muscles, which are essential for laryngeal elevation during swallowing. Various rehabilitative exercises have been developed to target these muscles, including tongue-strengthening [4], jaw-opening [5], and head-raising maneuvers such as the Shaker exercise [6]. However, the physical demands of the Shaker exercise often limit its feasibility, especially for frail or elderly individuals.

The Forehead Exercise for Suprahyoid Muscles (FESM, also known as Enge-Odeko-Taiso) [7] is a simpler modification of head-raising exercises. Performed in a seated position, FESM consists of isometric chin-tuck contractions against manual resistance applied to the forehead, involving minimal cervical movement. This design minimizes neck strain and enhances accessibility for individuals with limited physical capacity. Prior studies have shown that FESM can improve swallowing function [8], and it is now widely adopted in Japan as a practical substitute for the traditional Shaker exercise.

Both FESM and the Shaker maneuver facilitate anterosuperior movement of the hyoid bone and larynx, promoting upper esophageal sphincter (UES) opening and reducing pharyngeal residue [6,8]. While the long-term benefits of these exercises are well established [4-7], evidence regarding their immediate effects on swallowing remains limited. One report described immediate posterosuperior movement of the hyoid bone and elevation of the thyroid cartilage following a chin-pull maneuver in individuals with pharyngeal dysfunction [9]. However, the acute effects of FESM in patients with diagnosed dysphagia have not been well investigated.

We conducted a retrospective case series to evaluate the immediate impact of FESM on swallowing function in patients with dysphagia. Specifically, we examined whether performing FESM immediately prior to swallowing reduces the risk of penetration and aspiration, as assessed by the Penetration-Aspiration Scale (PAS) [10] during videofluoroscopic swallowing studies (VFSS).

## Materials and methods

Participants

We retrospectively reviewed patients who underwent VFSS at Hamamatsu City Rehabilitation Hospital between December 1, 2024, and April 1, 2025. Patients were eligible for inclusion if they demonstrated penetration or aspiration during VFSS, defined as a PAS score ≥2, and performed the FESM within the same VFSS session. Twelve consecutive patients met these criteria and were included in the analysis. No additional exclusion criteria were applied.

Intervention: forehead exercise for suprahyoid muscles (Enge-Odeko-Taiso)

**Figure 1:**
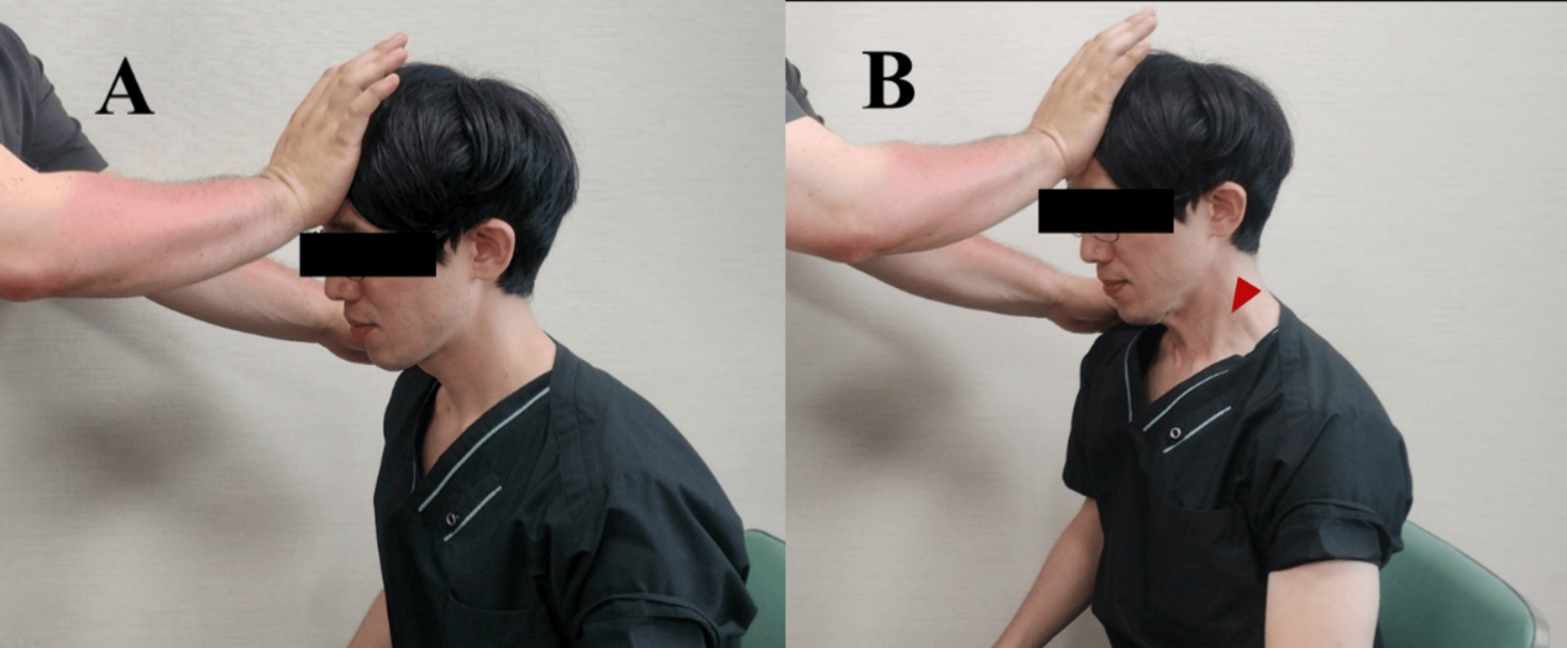
Forehead Exercise for Suprahyoid Muscles (FESM, Enge-Odeko-Taiso) A healthy volunteer looks toward the navel while applying manual resistance to the forehead, maintaining minimal cervical movement (isometric contraction). Participants were instructed to exert moderate to maximal effort during the contraction, depending on their physical capacity. (A) Rest position; (B) isometric contraction of the suprahyoid muscles while resistance is applied. The red arrowhead indicates visible cervical muscle contraction during the maneuver.

FESM was performed during VFSS under the supervision of a rehabilitation doctor and a speech-language-hearing therapist (Figure 1).

The decision to administer FESM was based on clinical judgment, typically following real-time observation of PAS ≥2 during VFSS. The institutional FESM protocol consists of five consecutive one-second isometric chin-tuck contractions (repetition mode) followed by a single five-second isometric chin-tuck contraction (holding mode).

Because of the retrospective nature of the study, detailed logs of exact repetitions and hold durations were not consistently available; however, every participant completed at least five repetitions of FESM in accordance with our institutional protocol. Because of these retrospective limitations, dose-response analyses were not performed. To ensure comparability, body position, bolus type, and imaging parameters were kept constant before and after the intervention.

Data collection and measurements

Demographic and clinical information was extracted from electronic medical records and VFSS reports. VFSS examinations were performed in the lateral projection with a Veradius Unity mobile C-arm system equipped with a flat-panel detector (Philips, Amsterdam, Netherlands) operating at 30 pulses per second and 30 frames per second. Recorded variables included age, sex, primary diagnosis, Food Intake Level Scale (FILS) score [11], reclining angle, and neck posture.

PAS scores were independently assigned by two rehabilitation physicians with more than five years of experience in swallowing assessment. When discrepancies arose, the video clips were jointly reviewed, and a consensus score was determined. The raters were aware of whether each video corresponded to a pre- or post-intervention swallow.

Statistical analysis

Descriptive statistics were used to summarize patient characteristics. Interrater reliability for PAS scoring was assessed using the weighted Cohen’s kappa coefficient. Pre- and post-FESM PAS scores were compared using the Wilcoxon signed-rank test. All statistical analyses were conducted using R (version 4.3.2; R Foundation for Statistical Computing, Vienna, Austria) and RStudio (version 2023.12.1+402; Posit Software, Boston, MA). A two-sided p-value of <0.05 was considered statistically significant.

Ethical considerations

This study followed the Strengthening the Reporting of Observational Studies in Epidemiology (STROBE) guidelines. Ethical approval was granted by the Ethics Committee of Hamamatsu City Rehabilitation Hospital (approval no. 25-03). All data were anonymized prior to analysis, and the requirement for informed consent was waived due to the retrospective nature of the study.

## Results

Twelve patients (nine males, three females; mean age: 76.6 ± 11.3 years) met the inclusion criteria. Baseline characteristics - including age, sex, primary diagnosis, FILS score, neck position, reclining angle, and food type - are summarized in Table 1.

**Table 1:** Individual Patient Characteristics and Pre-/Post-FESM Outcomes FESM = Forehead Exercise for Suprahyoid Muscles; FILS = Food Intake Level Scale; NA = not available; PAS = Penetration-Aspiration Scale

No.	Age (years)	Sex	Diagnosis	FILS	Neck position	Reclining angle	Food type	Aspiration timing (before FESM)	PAS (before FESM)	Aspiration timing (after FESM)	PAS (after FESM)
1	70	Male	Aspiration pneumonia	8	Neck flexion	90°	Mildly thick liquid from a cup	Before swallowing	8	NA	1
2	76	Male	Cerebral hemorrhage	8	Neck flexion	90°	3 mL of barium water	Before swallowing	3	NA	1
3	82	Male	COVID-19	8	Head rotation	60°	Barium water, from a cup	During swallowing	8	During swallowing	3
4	78	Female	Cerebral hemorrhage	9	Neck flexion	90°	Barium water, from a cup	Before swallowing	4	Before swallowing	2
5	77	Male	Cerebral hemorrhage	8	Neck flexion	90°	Crushed agar jelly mixed with mildly thick liquid	During swallowing	3	NA	1
6	85	Male	Cerebral infarction	7	Neck flexion	90°	Barium water, from a cup	During swallowing	2	NA	1
7	82	Male	Cerebral infarction	8	Head rotation	90°	Barium water, from a cup	During swallowing	8	NA	1
8	81	Female	Disuse syndrome	9	Neck flexion	90°	Barium water, from a cup	During swallowing	2	NA	1
9	88	Male	Cerebral infarction	8	Neck flexion	90°	3 mL of barium water	During swallowing	3	NA	1
10	77	Female	Cerebral infarction	9	Neck flexion	90°	Barium water, from a cup	Before swallowing	2	Before swallowing	2
11	44	Male	Cerebral hemorrhage	8	Neck flexion	90°	Barium water, from a straw	During swallowing	7	During swallowing	2
12	79	Male	Disuse syndrome	8	Neck flexion	90°	Barium water, from a cup	During swallowing	5	Before swallowing	8

Eight patients had cerebrovascular disease as the primary diagnosis. The median FILS score was 8.0 (interquartile range (IQR), 8.0-8.25), and all participants were consuming a full oral diet for all three meals. VFSS was conducted in a seated position for 11 patients and in a 60-degree reclined position for one. Neck posture was characterized by flexion in 10 patients and a combination of flexion and head rotation in the remaining two. Interrater reliability for PAS scoring was excellent. The weighted Cohen’s kappa coefficient was 0.97 (95% CI: 0.93-1.00), indicating a very high level of agreement between the two raters. This analysis was based on 24 VFSS videos (pre- and post-intervention) from 12 cases. Raters agreed on 22 of the 24 videos, with disagreement observed in two cases, resulting in a 91.7% raw agreement rate.

Penetration or aspiration was observed during test swallows using crushed agar, mildly thick barium [12], or barium water. The median pre-FESM PAS score was 3.5 (IQR: 2.75-8.00). Aspiration occurred before swallowing in four cases and during the swallow in eight.

**Figure 2:**
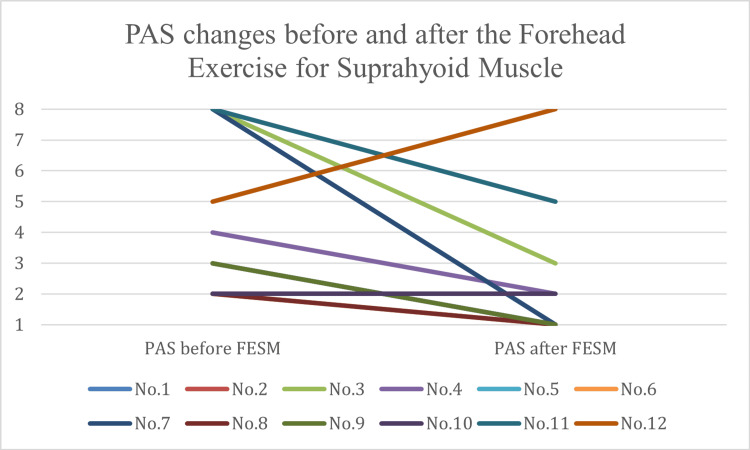
Changes in Penetration-Aspiration Scale (PAS) Scores Before and After the Forehead Exercise for Suprahyoid Muscles (FESM)

Following FESM, the median PAS score significantly decreased to 1.0 (IQR: 1.00-2.25), with a median improvement of 2.0 points (95% CI: 1.00 to 4.50; p = 0.025, Wilcoxon signed-rank test; Figure 2).

The effect size (r = 0.476) indicated a moderate to large impact. Of the 12 patients, 10 showed improvement, one showed no change, and one worsened.

In a representative case, the PAS score improved from eight before performing the FESM (Video 1) to one after performing the FESM (Video 2).

**Video 1:** Swallowing of Mildly Thick Liquid From a Cup Before Performing the Forehead Exercise for Suprahyoid Muscles (FESM)

**Video 2:** Swallowing of Mildly Thick Liquid From a Cup After Performing the Forehead Exercise for Suprahyoid Muscles (FESM)

## Discussion

This case series demonstrated that performing the FESM immediately before swallowing led to improved PAS scores during VFSS. To our knowledge, this is the first study to report the immediate effects of FESM, suggesting its potential as a simple and clinically feasible intervention for patients with dysphagia. While prior research has focused on the long-term effects of swallowing rehabilitation techniques such as the Shaker exercise and chin tuck against resistance (CTAR), their acute effects remain understudied. FESM is thought to be effective for the suprahyoid muscles, including the geniohyoid, mylohyoid, and anterior belly of the digastric muscles, similar to other exercises such as the Shaker exercise and CTAR. In contrast to the Shaker exercise, FESM involves less physical strain. Compared to CTAR, it does not pose a risk of temporomandibular joint (TMJ) pain or damage. Our findings indicate that FESM may serve as a novel therapeutic option with immediate benefits in reducing penetration and aspiration during oral intake or direct swallowing therapy.

The observed improvement in PAS scores likely reflects the effects of isometric contraction of swallowing-related muscles, particularly the suprahyoid group. Weakness in these muscles can impair laryngeal elevation and reduce pharyngeal contractility, thereby compromising airway protection. Exercises like the Shaker and FESM aim to strengthen these muscles and promote anterosuperior movement of the larynx, facilitating UES opening. Although such adaptations are typically reported as long-term outcomes, our results suggest that even a single session of FESM may elicit measurable biomechanical improvements.

Previous studies have shown that continued training of FESM increases geniohyoid muscle thickness [7], and similar isometric exercises, such as the chin push-pull maneuver, have demonstrated immediate elevation of the hyoid bone and thyroid cartilage [9]. Isometric contractions have also been reported to enhance muscle strength acutely [13], supporting the plausibility of short-term functional gains. One study found a significant increase in the percent change in thyrohyoid distance, measured from the onset of swallowing to peak anterior-superior hyoid excursion, after six weeks of Shaker exercise [14]. Given the biomechanical similarities between the Shaker and FESM, it is suggested that FESM also engages the thyrohyoid muscle. Improved thyrohyoid function may further enhance laryngeal elevation and closure, contributing to safer and more efficient swallowing. FESM may also promote an immediate anatomical elevation of the larynx. Although documented as a long-term adaptation to the Shaker exercise [14], a similar, transient response following FESM could explain the acute improvements observed in our study. Furthermore, post-activation potentiation, a phenomenon in which muscle performance is transiently enhanced following a voluntary conditioning contraction, could also play a role. This physiological response, characterized by increased force output and a faster rate of muscle activation, has been linked to mechanisms such as phosphorylation of myosin regulatory light chains, recruitment of higher-order motor units, and changes in pennation angle [15]. Although evidence in the field of dysphagia is limited, this mechanism may help explain the rapid effects of FESM on swallowing function and warrants further investigation.

In one case, the PAS score worsened, with aspiration occurring before, rather than during, the swallow. This may have resulted from premature bolus spillage due to impaired oral control, rather than a direct adverse effect of FESM. In this instance, the patient drank barium water from a cup. Although VFSS revealed no clear difference in sip volume, subtle variations in bolus size could not be entirely ruled out and may have contributed to the pre-swallowing aspiration. These findings suggest that FESM may be more effective in individuals with pharyngeal-phase dysfunction, rather than those whose aspiration arises from oral-phase impairment.

This study has several limitations. Although the sample size was relatively small and the study employed a retrospective, single-center design, a moderate to high effect size was observed, suggesting potential clinical relevance. Nevertheless, the limited number of cases and lack of a control group may affect generalizability and warrant cautious interpretation. In addition, FESM was applied based on clinician judgment during VFSS, introducing potential selection bias. Another limitation is the lack of blinding during PAS scoring, which may have introduced observer bias in the evaluation of pre- and post-intervention differences. The lack of standardized documentation also precluded analysis of dose-response relationships.

## Conclusions

This study demonstrated that a single session of FESM was associated with reduced penetration aspiration during swallowing in patients with dysphagia. The maneuver is quick, equipment-free, and well tolerated, making it feasible for routine bedside assessments and pre-meal protocols in both acute and long-term care settings. Its simplicity also enables caregiver-supervised home practice, potentially broadening therapeutic reach beyond the clinic. While findings are based on a small retrospective series, they highlight FESM as a promising, non-invasive preparatory exercise. Future studies with prospective designs, larger sample sizes, and control groups are warranted to validate the effectiveness of FESM and further explore its clinical applicability.

## References

[1] Park YS, Hong HP, Ryu SR, Lee S, Shin WS (2022). Effects of textured food masticatory performance in older people with different dental conditions. BMC Geriatr.

[2] Eltringham SA, Kilner K, Gee M, Sage K, Bray BD, Smith CJ, Pownall S (2020). Factors associated with risk of stroke-associated pneumonia in patients with dysphagia: a systematic review. Dysphagia.

[3] Wirth R, Dziewas R, Beck AM (2016). Oropharyngeal dysphagia in older persons - from pathophysiology to adequate intervention: a review and summary of an international expert meeting. Clin Interv Aging.

[4] Robbins J, Kays SA, Gangnon RE, Hind JA, Hewitt AL, Gentry LR, Taylor AJ (2007). The effects of lingual exercise in stroke patients with dysphagia. Arch Phys Med Rehabil.

[5] Wada S, Tohara H, Iida T, Inoue M, Sato M, Ueda K (2012). Jaw-opening exercise for insufficient opening of upper esophageal sphincter. Arch Phys Med Rehabil.

[6] Shaker R, Kern M, Bardan E (1997). Augmentation of deglutitive upper esophageal sphincter opening in the elderly by exercise. Am J Physiol.

[7] Ogawa N, Ohno T, Kunieda K, Watanabe M, Fujishima I (2024). A novel exercise to improve suprahyoid muscle area and intensity as evaluated by ultrasonography. Dysphagia.

[8] Sugaya N, Goto F, Seino Y, Nishiyama K, Okami K (2021). The effect of laryngeal elevation training on swallowing function in patients with dysphagia. J Laryngol Otol.

[9] Iwata Y, Nagashima K, Hattori T (2007). Investigation of training methods for patients with mild dysphagia. Jibi to Rinsho.

[10] Rosenbek JC, Robbins JA, Roecker EB, Coyle JL, Wood JL (1996). A penetration-aspiration scale. Dysphagia.

[11] Kunieda K, Ohno T, Fujishima I, Hojo K, Morita T (2013). Reliability and validity of a tool to measure the severity of dysphagia: the Food Intake LEVEL Scale. J Pain Symptom Manage.

[12] Matsuo K, Fujishima I (2020). Textural changes by mastication and proper food texture for patients with oropharyngeal dysphagia. Nutrients.

[13] Rio E, Kidgell D, Purdam C, Gaida J, Moseley GL, Pearce AJ, Cook J (2015). Isometric exercise induces analgesia and reduces inhibition in patellar tendinopathy. Br J Sports Med.

[14] Mepani R, Antonik S, Massey B (2009). Augmentation of deglutitive thyrohyoid muscle shortening by the Shaker Exercise. Dysphagia.

[15] Tillin NA, Bishop D (2009). Factors modulating post-activation potentiation and its effect on performance of subsequent explosive activities. Sports Med.

